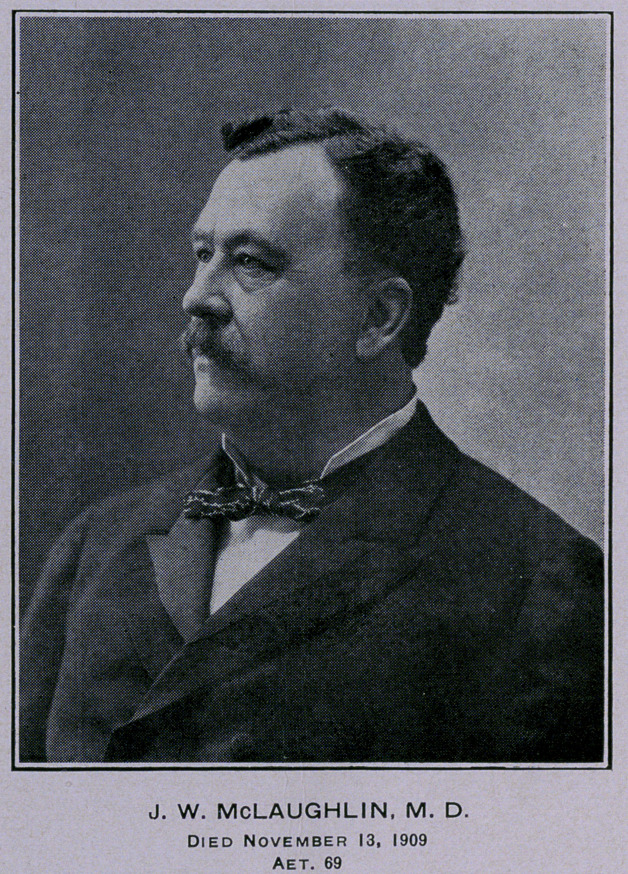# Life, Character and Works of Prof. J. W. McLaughlin, M. D.*Delivered by invitation at the annual convention of the Texas State Medical Association at Dallas, Texas, May 11, 1910.

**Published:** 1910-06

**Authors:** H. L. Hilgartner

**Affiliations:** Austin, Texas


### TEXAS MEDICAL JOURNAL.

Established July, 1885

F. E. DANIEL, M. D., - - - - - - Editor, Publisher and Proprietor

Published Monthly.—Subscription $1.00 a Year.

VOL. XXV AUSTIN, JUNE, 1910. No. 12

The publisher is not responsible for the views of the contributors.

### Original Articles.

### Life, Character and Works of Prof. J. W. McLaughlin, M. D.*

BY H. L. HILGARTNER, M. D., AUSTIN, TEXAS.

*Mr. President, Members of the Texas State Medical Association, Ladies and Gentlemen:*

By the untimely death of Dr. J. W. McLaughlin, on November 13, 1909, under circumstances of peculiar sadness, and at a comparatively early age, we lost, I am sure we would all agree, the foremost physician in the State.

It has been said that "the history of an epoch is the history of its leading men. They are the center and source of intellectual energy. In them the ever-widening waves of mental progress have their origin, and it is under their vivifying influence that science and learning grow and spread."

We, therefore, do well, from time to time, to gauge our gains by contemplating the life work of the men who have largely contributed to them, and have thus left behind the imprint of their power and their individuality. It is considerations such as these that induce the Texas State Medical Association to see in these occasions, brought to our notice by the stern act of death, opportunities, not only for showing our esteem and admiration for those who have wrought faithfully and well, but also for dwelling for a

*Delivered by invitation at the annual convention of the Texas State Medical Association at Dallas, Texas, May 11, 1910.

too brief moment upon their life history and estimating albeit imperfectly the worth of their labors and their lives.

As a friend of nearly twenty years, who enjoyed a rarely privileged intimacy for more than half that time, I have charged myself with the sad but cherished duty of pronouncing a few words in honor of the memory of my friend. That my effort will prove but feeble I know only too well. It would require a man of broad learning and real power, a gifted orator and a master of our English speech, to do justice to the life and works of J. W. McLaughlin.

Born near Springfield, Ohio, the 7th day of September, 1840, on the death of his father, C. D. McLaughlin, the subject of this sketch, to use his own words, "engaged in the study of medicine with his uncle, Dr. A. C. McLaughlin, with whom he lived until the breaking out of the war between the States in April, 1861. Being an earnest and ardent supporter of State's rights, the Wide-Awakes soon convinced young McLaughlin that south of Mason and Dixon's line was his only safe domicile, and, this move being in perfect harmony with the political views of the young man, he quietly disappeared from his home early in April, 1861, between sunup and sundown. Reaching Louisville, Ky., he enlisted in Company D, First Kentucky Infantry, and at Harper's Ferry was sworn into the Confederate Army to serve for twelve months. He remained in the service, however, until the army surrendered in 1865, serving at various times under Johnston, Jackson, Morgan and Forrest.

"Being still rebellious and unreconstructed, McLaughlin and A. H. Cross, an army comrade, started for South America. But on reaching Texas, McLaughlin met Miss Tabitha Bird Moore, who later became his wife, and decided to take up medicine as a profession. He at once entered into its practice with Dr. Sam D. McLeary, near Columbus, Texas. Working diligently, the following spring (1867), after completing the prescribed course of study, he graduated from the Medical Department of the University of Louisiana.

"His marriage was solemnized in September of the same year and with his devoted wife he located in Fayette county for the practice of his profession, where he remained until 1869, when he removed to Austin."

Dr. McLaughlin practiced in Austin thirty-two years, when he was called to the Chair of Medicine in the Medical Department of the University of Texas. This chair he filled with distinction eight years, when he resigned and returned to Austin. Dr. McLaughlin was President of the Texas State Medical Association in 1894, a Regent of the University of Texas from 1907 until his death, and President of the Texas Academy of Science at the time of his death.

The brief reference to Dr. McLaughlin's war record, that time will permit, can not be better made than in the words spoken by Col. Joel H. B. Miller by the side of his grave.

"Comrades," he said, "the Confederate soldiers who took up arms to repel the invasion of the Southern States were patriots. But natives, men to the manner born, would have been recreant to their duty if they had failed to defend the institutions of their land, its fundamental principles of local self-government, their women and their children, their homes and their firesides. It was patriotism that fired the hearts of Southern men in the Civil War, but we must confess that there was a loftier and more disinterested patriotism. Every instinct of the Southern man was aroused to its highest. By education, by environment, by all that made home sacred, his heart beat to the call to arms. But there was a more disinterested and higher patriotism and it was exhibited by our comrade James W. McLaughlin. He was born and reared under a Northern sky, his associations were Northern, and all his ties were to the institutions of the North. But he recognized that there was a basic principle involved in the war, an issue that went to the very foundation of civil government, and he sacrificed all of his life-long associations, all of his local opportunities, friends and home to unite with us in defence of that principle. He abandoned all of that. As a stranger in a strange land he came South to offer his services to a government that maintained that principle.

"He joined the boldest and most brilliant of our cavalry commands and fought in the ranks, shoulder to shoulder, with those who wore the gray. He was one of the bravest and most daring that followed that gallant soldier, John Morgan, and he won his spurs in the front rank of that brilliant and dashing command. He fought on our side during the war and became enamoured of Southern chivalry and Southern manners; and at the conclusion of the war he settled in the South to practice a profession, that of medicine, that offered the largest field for his splendid intellect, and most humane sympathy. And with him peace had victories as great as war. He soon became one of the most distinguished in his profession, and his skill and gentle presence in the sick-room made that presence an encouragement and assurance to the sufferers to whom he so willingly ministered. He became not only a citizen of the South, but he worshiped with you and me on the sacred altars of the Confederacy. It is the glory of the South to have had such men, and their memories will live as an example to the youth of the land. He was our comrade in war and our physician and friend in peace, and we will cherish his memory as will those who come after us. His high character and goodness he carried with him to a better world, where his comrades will soon join him."

As a mature man the greater part of Dr. McLaughlin's energies were devoted to the practice of his profession. Of that I shall speak later, but the attraction of the scientific side was very strong and soon led him to devote much time and thought to the scientific work he liked best. Very early in his medical career his thoughts were directed toward an explanation of drug action. This led to investigations which resulted in his well-known wave-interference theory of catalysis, and catalytic theory of immunization, which he worked upon up to his last days. His views on the different problems constitute, in the judgment of the competent, a noteworthy contribution to scientific theory.

Possibly even more valuable was the work he did during the epidemic of dengue in 1885, which resulted in his discovery of the bacillus of that fever. When we remember that this work was done, not in a laboratory equipped with all modern appliances, which do so much to ease the work of the student and so little to develop his resourcefulness, but in a room at his residence, surrounded by the most meager appliances, we begin to appreciate his remarkable skill and ability as well as the exceptional strength of his devotion to the cause of science.

In 1887 he published an article entitled "The Etiology of Acute Infectious Diseases," in Daniel's TEXAS MEDICAL JOURNAL. In 1890 he read a paper at the Texas State Medical Association entitled "An Explanation of the Phenomena of Immunity and Contagion, Based Upon the Action of Physical and Biological Laws." In connection with the paper he said: "I found it impossible to intelligently and fully include a subject so complex and novel within the compass of a society essay, consequently the paper was seriously crippled by its brevity. Notwithstanding this defect, it received from some of the leading medical journals of this country very favorable notices, and the complete article was translated into a foreign language and published in a foreign medical journal. The encouragement I received from such favorable notice induced me to be more fully elaborate, and again publish an article on this subject. This I did during the last year in serial numbers of the *Texas Sanitarian.*"

These articles, corrected and enriched with new matter bearing upon this subject, compose the volume entitled "Fermentation, Infection and Immunity," published in 1892.

In addition to the above, I beg to mention several of his most important minor publications, as follows:

"The Advantages and Use of the Microscope to the General Practitioner in Diagnosis and Therapy." Read before the State Medical Association, April, 1904.

"Gastro-Intestinal Conditions in Epilepsy." Reprinted from the *Medical News* (New York), April 15, 1905.

"The Modern Diagnostic Methods in Cancer of the Stomach." Read before the State Medical Association, April, 1906.

"Personal Observation in Latent Malaria." Read before the Twelfth District Medical Society, January, 1908.

"A Catalytic Theory of Infection and Immunity." Reprinted from the *Medical Record,* May 1, 1909.

"Critical Remarks on Ehrlich's Side Chain Theory of Immunity." Presidential address of the Texas Academy of Science, October, 1909.

One of the most fruitful periods of Dr. McLaughlin's life was passed during the time he was Professor of the Practice of Medicine in the Medical Department of the University of Texas. I have received a long and appreciative letter from Dr. John T. Moore, who was his assistant during a large portion of that time, and from it I take the liberty of quoting freely, paraphrasing some parts of the letter in order not unduly to extend the time of this address.

On assuming the duties of this chair, Dr. McLaughlin saw at once the necessity of bringing the laboratory work of the students into close connection with the teaching in the hospital wards. Previously the laboratory had been in a building at a distance from the hospital. Towards this great improvement in methods he labored earnestly and enthusiastically, but with his usual unselfish and modest methods. For a time he and his assistant fitted up a room in the basement of the hospital, with simple apparatus, at their own expense, and it was only after bearing this burden for some years that the Regents of the University were able to provide funds for the equipment of a satisfactory laboratory. Dr. McLaughlin's method of securing this appropriation was characteristic of the man. He induced the Regents, during one of their meetings at Galveston, to visit his little laboratory, which seemed little more than a small room with a few inconspicuous instruments. After their entrance, the doctor began to explain the advantages of a modern laboratory, but soon one of the Regents interrupted him and asked to be shown this laboratory. Then the doctor, in his gentle way, said: "Gentlemen, you are now in the Clinical Laboratory of the University, and you behold all of our splendid equipment."

I am brought to the last and highest point of view in considering the place and worth of any man. I have spoken of J. W. McLaughlin as a soldier, as a scientist and as a doctor; it remains to speak of him as a man. But at this point especially do I feel not merely my own oratorical weakness, but the absolute inadequacy of words to describe a truly magnanimous man. Personal affection and bereavement also here tongue-tie me.

I can only bear witness that J. W. McLaughlin was a true man, perfect in all the "weightier matters" of character, justice, mercy and truth. One of the sincerest, naturally truest and most modest of men. He was free from every tinge of vanity or other petty feeling. I never saw a man who thought so little about himself or his own concerns. His temper was imperturbably good, with the most winning and courteous manners; yet, as I have seen, he could be aroused by any bad action to the warmest indignation and prompt measures. No one could be admitted to intimacy with him without enlarging ideals of courage and generosity—of all that we mean by manhood.

Never did he show more nobly than as I saw him day by day through the last months when relentless disease had prostrated even his enormous strength. Physical pain and weakness, however, could not impair his thoughtfulness for others and perfect courtesy, or abate his interest in the scientific problems upon which his thoughts and conversation dwelt to the very end:

"For he preserved from chance control
The fortress of his 'stablished soul,
In all things sought to see the whole,
Brooked no disguise,
And set his heart upon the goal,
Not on the prize."

His diligence and patient zeal in the search for the truth could not be more touchingly illustrated than by an episode of the last week of his sickness. We had a mutual friend well qualified to discuss the scientific questions in which Dr. McLaughlin was most interested. Several letters dealing with certain points of such discussion had passed between them. Finally, about six weeks before his death, McLaughlin penciled with his own hand, as he lay on his bed of suffering, fourteen closely written pages which he gave me to mail to our friend. I take the liberty of quoting the concluding paragraph of the letter to Dr. McLaughlin sent in response to that paper. After highly appreciative remarks upon the doctor's statements from a scientific point of view, the writer added:

"But, dear Dr. McLaughlin, it is the fact that *you* should have taken the trouble to write and send the exposition to me which moves me to an admiration of your generosity and to a warmth of response to the friendship with which you honor me that could not be described in words. It is with especially poignant sorrow, therefore, that I have heard of the severity of your sickness. I hope it is only a passing attack; but in any case there is naught to say to a man of your magnanimity except the unvoiced meaning of a hand-clasp offered by one who loves and honors you. Our intercourse has been limited, but I have always had the sense to recognize a great mind and noble heart whenever the commanding vision of that rare existence has been vouchsafed me. You are one of a very few men whom to have known in the noble mystery of friendship's election I count the best element in my own being, and the ground for an otherwise dubious hope of some worthiness in myself."

Even as through those long days of sickness I had no way to express my sorrow for our approaching separation and my admiration for his great mind and noble heart, except by the "unvoiced meaning of a hand clasp," so now is seems to me that for those who knew him words would be superfluous, and for those who knew him not it is too late.

On the other hand there is a truth in the poet's exclamation:

"The living do not rule this world. Ah, no!
It is the dead—the dead."

For the good and great do leave an abiding influence if those who saw and *knew* pass the vision onward.

So I have stood here before you, not to stammer an eulogy in empty words, but to pay my tribute of loyalty to a great man and to his ideals, for

"There are deeds that should not pass away,
And names that must not wither."

## Figures and Tables

**Figure f1:**